# Underexplored reciprocity between genome-wide methylation status and long non-coding RNA expression reflected in breast cancer research: potential impacts for the disease management in the framework of 3P medicine

**DOI:** 10.1007/s13167-023-00323-7

**Published:** 2023-05-22

**Authors:** Andrea Kapinova, Alena Mazurakova, Erika Halasova, Zuzana Dankova, Dietrich Büsselberg, Vincenzo Costigliola, Olga Golubnitschaja, Peter Kubatka

**Affiliations:** 1 Biomedical Center Martin, Jessenius Faculty of Medicine, Comenius University in Bratislava, 036 01 Martin, Slovakia; 2 Department of Anatomy, Jessenius Faculty of Medicine, Comenius University in Bratislava, 036 01 Martin, Slovakia; 3 Weill Cornell Medicine-Qatar, Education City, Qatar Foundation, 24144 Doha, Qatar; 4 European Medical Association, EMA, Brussels, Belgium; 5 Predictive, Preventive, and Personalised (3P) Medicine, Department of Radiation Oncology, University Hospital Bonn, Rheinische Friedrich-Wilhelms-Universität Bonn, 53127 Bonn, Germany; 6 Department of Medical Biology, Jessenius Faculty of Medicine, Comenius University in Bratislava, 036 01 Martin, Slovakia

**Keywords:** Breast cancer, Epigenetic modifications, Methylation, Non-coding RNAs, lncRNAs, Biomarker panels, Predictive preventive personalized medicine (PPPM/3PM), Pre/clinical studies, Primary and secondary care, Suboptimal health, Mitochondria, Health-to-disease transition, Phenotyping, Homocysteine, Endothelin-1, Metastatic disease, Population screening, Improved healthcare economy, Health policy

## Abstract

Breast cancer (BC) is the most common female malignancy reaching a pandemic scale worldwide. A comprehensive interplay between genetic alterations and shifted epigenetic regions synergistically leads to disease development and progression into metastatic BC. DNA and histones methylations, as the most studied epigenetic modifications, represent frequent and early events in the process of carcinogenesis. To this end, long non-coding RNAs (lncRNAs) are recognized as potent epigenetic modulators in pathomechanisms of BC by contributing to the regulation of DNA, RNA, and histones’ methylation. In turn, the methylation status of DNA, RNA, and histones can affect the level of lncRNAs expression demonstrating the reciprocity of mechanisms involved. Furthermore, lncRNAs might undergo methylation in response to actual medical conditions such as tumor development and treated malignancies. The reciprocity between genome-wide methylation status and long non-coding RNA expression levels in BC remains largely unexplored. Since the bio/medical research in the area is, per evidence, strongly fragmented, the relevance of this reciprocity for BC development and progression has not yet been systematically analyzed. Contextually, the article aims at:

- consolidating the accumulated knowledge on both—the genome-wide methylation status and corresponding lncRNA expression patterns in BC and
- highlighting the potential benefits of this consolidated multi-professional approach for advanced BC management.

Based on a big data analysis and machine learning for individualized data interpretation, the proposed approach demonstrates a great potential to promote predictive diagnostics and targeted prevention in the cost-effective primary healthcare (sub-optimal health conditions and protection against the health-to-disease transition) as well as advanced treatment algorithms tailored to the individualized patient profiles in secondary BC care (effective protection against metastatic disease). Clinically relevant examples are provided, including mitochondrial health control and epigenetic regulatory mechanisms involved.

## Introduction

### Why the paradigm change from reactive medicine to 3 PM approach is essential for breast cancer research and overall disease management?

According to the current statistics, breast cancer (BC) represents the most frequently diagnosed type of malignancy with the highest mortality (mainly due to the BC-associated metastatic disease) in women worldwide [1, 2]. Despite improved early screening and more effective therapeutic strategies, more than 90% of BC mortality is attributable to advanced or metastatic disease [3–5]. Therefore, a better understanding of cellular and molecular mechanisms regulating BC cell plasticity [6] is crucial to improve overall BC management reflected in innovative PPPM strategies comprising individualized patient profiling, predictive diagnostics, targeted prevention in primary and secondary care, and treatment algorithms tailored to the person—altogether leading to improved individual outcomes and healthcare economy [7, 8]. To this end, epigenetic regulation plays a pivotal role in the interplay between genotype and phenotype and promoting and protection against the health-to-disease transition, which is instrumental for the paradigm change from reactive care to predictive, preventive, and personalized approach [9].

### Evidence towards reciprocity between genome-wide methylation and long non-coding RNA expression levels

**Fig. 1:**
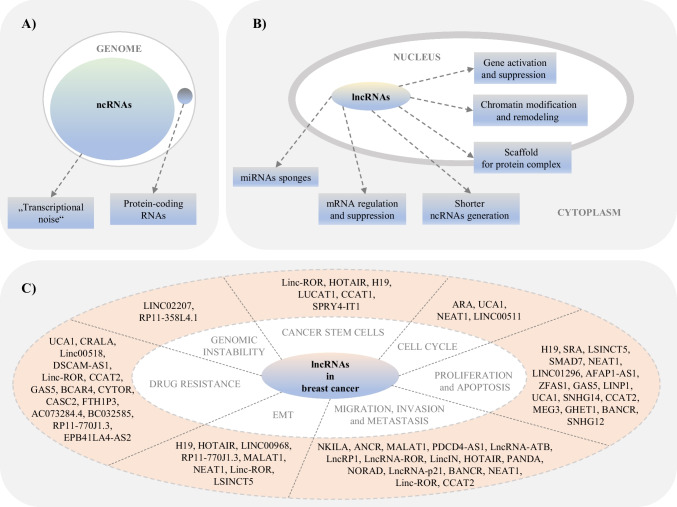
**A** Genome vs transcriptome, and ncRNAs vs protein-coding RNAs. **B** Essential functions of lncRNAs in the cell. **C** The roles of lncRNAs in breast cancer. **A** Most of the mammalian genome is actively transcribed. However, non-coding RNAs, formerly called “transcriptional noise” or “junk,” form a substantial part of the transcriptome. Besides, less than 2% of the transcripts code for proteins. **B** Long non-coding RNAs forming the most prevalent and diverse class of regulatory ncRNAs are linked to different cellular functions, including gene activation, chromatin modification and remodeling, scaffold for protein complex, shorter ncRNAs generation, mRNA regulation and suppression, and miRNA sponges. **C** Numerous lncRNAs participated in regulating different stages of breast cancer, for example, cell cycle progression, proliferation and apoptosis, migration, invasion, metastasis, EMT, drug resistance, genomic instability, or breast cancer stem cells. LncRNAs acted as either promoters or inhibitors of the abovementioned key processes associated with breast carcinogenesis [15, 16, 21–32]

The study of biological functions of the human genome encoding non-coding RNAs (ncRNAs) is a subject of intensive scientific research nowadays (Fig. 1A). Commonly, NcRNAs are defined as RNAs with no protein-coding potential but with a proven significant regulatory role in various physiological and pathological processes, including cancer, at the epigenetic, transcriptional, or post-transcriptional level [10]. Long non-coding RNAs (lncRNAs) represent one of the largest and most diverse classes of ncRNAs. Although the research of lncRNAs is still in infancy, and there is a large gap between the number of existing lncRNAs and their known relation to molecular or cellular function, lncRNAs are considered master gene regulators at the epigenetic level in carcinogenesis (Fig. 1B). An improved understanding of the function and role of lncRNAs in cancer is therefore highly required. In the context of BC, current studies have shown that specific dysregulation of many lncRNAs is associated with the development and progression of BC and significantly correlates with poor outcomes in BC patients (Fig. 1C) [11–18]. These discoveries suggest that lncRNAs could represent suitable diagnostic and prognostic biomarkers and potential therapeutic targets for BC management. However, these conclusions are mainly based on the study of aberrant lncRNA expression profiles, while the regulatory mechanisms, e.g., methylation patterns, conditioning these changes remain largely unexplored. Most recent BC mechanistic evidence suggests several plausible mechanisms. Firstly, lncRNAs can regulate the methylation status of DNA, RNA, and histones. Secondly, the methylation status of DNA, RNA, and histones can affect lncRNAs levels. Third, lncRNAs may undergo methylation in response to medical conditions such as tumor development and treated malignancies [19, 20].

### Aims of the study in the framework of predictive, preventive, and personalized medicine

Both genetic and epigenetic alterations cause BC development and disease progression. Previous studies focused mainly on identifying methylation patterns of protein-coding genes concerning BC. However, increasing evidence indicates a potential functional interplay between genome-wide methylation status and expression levels of lncRNAs. The latter is considered a hub in regulating epigenetic events highly relevant for BC development and BC progression into metastatic disease. Contextually, the current review article aims at consolidating the accumulated knowledge on genome-wide methylation status in correspondence with lncRNA patterns with their reciprocity relevance, specifically for BC in research and healthcare. The proposed approach may be of clinical potential to benefit affected patient cohorts and disease-predisposed individuals. The results could strongly contribute to BC prediction, development of innovative screening programs, targeted prevention, treatment algorithms tailored to the person, and improved individual outcomes and overall economy of BC management.

Here, we hypothesize that highlighted innovative biomarker panels are of potential clinical utility in primary (sub-optimal health conditions and protection against the health-to-disease transition) and secondary (adequate protection against metastatic BC) care.

### Source of data

English-language biomedical literature sources from PubMed bibliographic database were collected and analyzed for the topic-related items, including all the keywords or medical subject heading (MeSH) terms listed above. The most recent scientific publications from 2017 to 2023 were mainly considered for the final statement presented in this paper.

## Genome-wide methylation profiling and its correlation with lncRNA expression and carcinogenesis

**Fig. 2:**
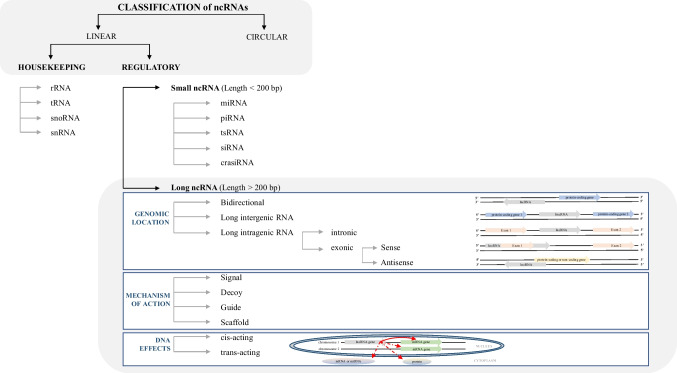
Schematic representation of a classification of non-coding RNAs based on their structure, function, length, genomic location, mechanism of action, and effects on DNA, emphasizing long non-coding RNAs; NcRNAs can be divided into linear or circular. According to their function, ncRNAs are recognized as housekeeping or regulatory. Housekeeping ncRNAs are constitutively expressed in each cell type, required for their viability and primarily regulating generic and essential functions of cells. The regulatory ncRNAs act as key regulators of various RNA molecules and gene expression at the epigenetic, transcriptional, and post-transcriptional levels. Based on their length, ncRNAs can be divided into small or long. LncRNAs can be genomically located between two protein-coding genes (intergenic lncRNAs), in an intron of a coding region (intronic lncRNAs), or within 1 kb of promoters and transcribed from the same promoter as a protein-coding gene yet in the opposite direction (bidirectional lncRNAs). Other lncRNAs can be transcribed either from the sense RNA strand of the protein-coding genes (sense lncRNAs) or the antisense RNA strand of a protein-coding gene (antisense lncRNA) might overlap one or several introns and/or exons. According to the mechanism of action, lncRNAs can be divided into four groups—signal, decoy, guide, and scaffold. Signal lncRNAs, with regulatory function, are expressed at a specific time and in a particular position in the cell as a response to stimuli. Signal lncRNAs can mediate the transcription of downstream genes alone or in combination with other proteins. Decoy lncRNAs can indirectly repress transcription, either binding to some functional proteins and blocking them from regulating DNA and mRNA or binding to miRNA competitively with mRNA and blocking the inhibitory effect of miRNA on mRNA. Guide lncRNAs are necessary to organize and locate some functional proteins at specific genomic loci to perform their functions. Scaffold lncRNAs are important in assembling multi-protein complexes in the target area. Moreover, lncRNAs can mediate epigenetic regulation via chromatin-modifying proteins in cis or trans manner. Cis-acting lncRNAs affect target genes located near the lncRNA gene on the same chromosome, while trans-acting lncRNAs affect target genes situated distal to the lncRNA gene, often in a different chromosome [22, 24, 26, 28, 30–32, 49–51]; Abbreviations used: crasiRNA, centromere repeat associated small interacting RNA; miRNA, microRNA; ncRNAs, non-coding RNAs; piRNA, piwi RNA; rRNA, ribosomal RNAs; siRNA, small interfering RNA; snoRNA, small nucleolar RNA; snRNA, small nuclear RNA; tRNA, transfer RNA; tsRNA, tRNA-derived small RNAs

NcRNAs (both groups—housekeeping ncRNAs and regulatory ncRNAs) can be genetically or epigenetically regulated (Fig. 2). Many diverse genetic variations affecting ncRNAs have been identified regarding carcinogenesis [21, 33–44]. Unlike genetic changes, epigenetic modifications represent heritable reversible changes that affect gene activity without changing the DNA and RNA sequence [45–47]. These modifications related to various DNA, histone, and chromatin modifications and changes in the regulation of ncRNAs play an essential role in different biological and pathological processes, including cancer [48].

LncRNAs are RNA transcripts above 200 bp long (i.e., their size varies from hundreds of base pairs to tens of kilobases) without open reading frames, characterized by complicated structures and intrinsic origins, typically expressed in a tissue-specific manner [52, 53]. LncRNAs possess mRNA-like characteristics, 5′ cap, and 3′ poly(A) tail but lack protein-coding ability [54]. Moreover, lncRNAs can interact with other RNAs, DNA, and proteins and thus affect almost every aspect of gene regulation. Furthermore, lncRNAs can serve as the precursor of various small RNAs, e.g., miRNAs, snoRNAs, and piRNAs, and regulate their expression and function [22, 55, 56]. Within the cells, lncRNAs are found in the nuclei, cytoplasm, or mitochondria. In the nucleus, lncRNAs can regulate chromatin re-modeling, transcription, translation, and mRNA turnover in the cytoplasm. Moreover, lncRNAs can pass from one cellular component to another, e.g., in dependence on environmental changes (Fig. 1B) [5, 57]. The first lncRNA H19 was discovered and characterized in 1990 [58]. Since then, the number of mammalian non-coding transcripts has spectacularly increased, and to date, according to the NONCODE database (Current Version v6.0), there are 173,112 human lncRNAs transcribed from 96,411 genomic loci [19, 59]. These lncRNAs are classified according to their origin in the genome into 6 groups: (1) sense lncRNA, (2) anti-sense lncRNA, (3) bidirectional lncRNA, (4) intronic lncRNA, (5) intergenic lncRNA, and (6) sense-overlapping lncRNA (Fig. 2). LncRNAs can regulate the major pathways leading to cancer development and progression at the epigenetic, transcriptional, or post-transcriptional level [5, 14, 60–64]. Specifically, lncRNAs can regulate critical genes involved in malignant transformation and either increase the activation of oncogenes or limit the expression of tumor-suppressor genes. Moreover, many lncRNAs are expressed in cell-type-, tissue-, disease-, or developmental stage-specific manner [65–69]. From an epigenetic point of view, lncRNAs are recognized as significant epigenetic regulators in carcinogenesis. Cancer research has shown that lncRNAs can regulate DNA, RNA, or histones methylation. On the contrary, the methylation status of DNA, RNA, and histones can affect the expression level of lncRNAs. Moreover, lncRNAs themselves can also be subject to the process of methylation.

### DNA methylation

DNA methylation is the most widely studied epigenetic alteration known since 1948, with a proven significant impact on the development of cancer and other diseases [54, 70–75]. DNA methylation is characterized by adding a methyl group (CH_3_) from S-adenyl methionine (SAM) onto the C5 position of the cytosine residue to form 5mC. DNA methylation is regulated by enzymes called DNA methyltransferases (DNMTs), also known as “writers.” Three members of DNMTs—DNMT1, DNMT3a, and DNMT3b—DNA methylation can be removed by enzymes known as demethylases, also referred to as “erasers.” Demethylases include TET enzymes (ten-eleven translocation methylcytosine dioxygenases). Lastly, DNA methylation can be recognized by three families of proteins, so-called readers, which include (1) the MBD proteins (containing a methyl-CpG-binding domain); (2) the UHRF proteins (ubiquitin-like, containing PHD, and RING finger domains); and the zinc-finger proteins (containing a zinc-finger domain) [19, 20, 76, 77]. In mammals, DNA methylation is typically found in CpG dinucleotides. About 80% of CpG sites are estimated to be methylated, excluding specific regions called CpG islands (CGIs). CGIs primarily exist in the promoter regions of genes. Therefore, methylation changes (hyper- or hypo-methylation) in the promoter regions of genes can be associated with alterations in their expression, either upregulation or downregulation [19, 20, 71]. Current research has shown that differences in DNA methylation profiles between normal and malignant tissues have the potential to serve as a diagnostic and/or prognostic marker in various types of cancer, including BC [72, 73]. Moreover, the interplay between DNA methylation and lncRNAs represents a critical layer of epigenetic regulation in carcinogenesis. DNA methylation mediated by lncRNAs can be crucial in tumor progression, proliferation, invasion, and metastasis of various tumor cells [19, 20, 78–80]. On the contrary, DNA methylation can affect the expression and function of multiple lncRNAs with a significant impact on the process of carcinogenesis [81, 82].

### Histone methylation

Histone methylation is another essential regulatory epigenetic mechanism closely associated with cancer development. Histone methylation mainly occurs on lysine and arginine residues within proteins. Lysine can be monomethylated (me1), dimethylated (me2), or trimethylated (me3). Arginine can be me1 or me2 symmetrically (me1s, me2s) or me2 asymmetrically (me2a) [83, 84]. Histone methyltransferases (HMTs) contain three group members. The first one consists of the SET domain and lysine methyltransferases (KMTs) (except of DOT1L (KMT4)), the second one consists of a non-SET domain and DOT1L and the PRDM protein family members with N-terminal PR domain, and the last one represents PRMT family that shares a common methyltransferase domain. Eight KDM families are known among histone demethylases (HDM) [85, 86]. Moreover, histone methylation can be recognized by various histone methylation readers, such as PWWP, chromodomain, PHD, Tudor, and WD40 [87]. Above all, DNA and histone methylation regulate chromatin structure and function synergistically [88]. Cancer research confirmed that lncRNAs could regulate histone methylation via involvement in the recruitment of polycomb proteins or methyltransferases associated with histone methylation of specific targets. On the contrary, histone methylation can affect lncRNAs and cause their activation or repression [89–92].

### RNA methylation modifications

According to the MODOMIC database, more than 170 kinds of RNA modifications have been confirmed so far [93]. RNA modifications affect all bases of RNA and the ribose moiety and are, therefore, more diverse and complex when compared to DNA modifications. Moreover, RNA modifications can occur in a highly dynamic fashion, thereby increasing the complexity of the RNA species on different levels, such as biogenesis, localization, structure, and function of RNAs [94–96]. Approximately 60% of RNA modifications represent methylated modifications [97]. RNA methylation is a reversible post-transcriptional RNA modification found in various coding and non-coding RNA types, including lncRNAs [98–100]. LncRNAs themselves can either undergo various methylation modifications or participate in developing some of them within the process of carcinogenesis. In general, RNA methylation (analogously like DNA methylation) is characterized by the transfer of CH_3_ from the cofactor (e.g., S-adenosyl-L-methionine) by methyltransferases to RNA molecules. Furthermore, RNA methylation can be removed by the demethylases and recognized by RNA-binding proteins (“readers”) [19, 20, 101]. Although RNA methylation is a relatively newly discovered mechanism of epigenetic regulation of gene expression, increasing evidence has revealed its crucial roles in signaling pathways regulating physiological and pathological processes. RNA methylation is involved in many aspects of RNA metabolism and is associated with regulating RNA splicing, translation, stability, degradation, translocation, function, and high-level structure [101, 102]. Within the process of carcinogenesis, RNA methylation represents a so-called dual-edged weapon. On the one hand, RNA methylation can act as an activator and trigger the carcinogenesis process (by promoting the expression of oncogenes or by inhibiting the expression of TS genes). But on the other hand, RNA methylation can act as an inhibitor or suppressor of carcinogenesis (by promoting the expression of TS genes or by inhibiting the expression of oncogenes) [19, 20, 103–105]. The development of epi transcriptomic methodologies (e.g., analyses used anti-modification antibodies or chemical methods coupled to the RNA methylation sequencing technology (NGS)) enabled to find and study several types of RNA methylations, such as N6-methyladenosine (m6A), N1-methyladenosine (m1A), and 5-methylcytosine (m5C), among others briefly mentioned below. There are also known other types of RNA modifications dysregulated in human cancers such as, e.g., 5-hydroxymethylcytosine (hm5C), 5-hydroxymethyl-2′-O-methylcytidine (hm5Cm), 5-methoxycarbonylmethyl-2-thiouridine (mcm^5^s^2^U), 5-methoxycarbonylmethyluridine (mcm^5^U), pseudouridine (Ψ), or adenosine-to-inosine (A-to-I) RNA editing [100, 106]. However, these RNA modifications are beyond the scope of the interest of this review (not purely methylation modifications), and therefore, we do not discuss them further below. Besides, m6A is the most intensely researched epigenetic RNA modification with a significant effect on carcinogenesis. However, the research on other types of RNA methylations is still not systematic and in-depth, probably owing to the difficulty in mapping this modification on the transcriptome [99, 107]. Further studies in RNA methylations are needed and highly required, especially in the context of BS as the most frequently diagnosed type of cancer nowadays.

#### N6-methyladenosine

N6-Methyladenosine (m6A), first identified in 1974 on messenger RNA (mRNA), represents one of the most common, dynamic, and deeply researched epigenetic modifications found in different types of eukaryotic coding as well as non-coding RNAs, including lncRNAs. M6A is assumed to occur in ~ 30% of all transcripts [19, 20, 101, 102, 105, 108]. m6A modification arises by methylation of the 6th nitrogen atom of adenine in RNA. Besides, m6A primarily mediates post-transcriptional regulation of gene expression by modifying RNA structure or specific binding [19, 20, 93, 109]. Importantly, m6A is modified by m6A methyltransferases (METTL3/14/16, RBM15/15B, ZC3H3, KIAA1429 (VIRMA), CBLL1, or WTAP), removed by m6A demethylases (FTO and ALKBH5 or ALKBH3), and recognized by m6A-binding proteins (YTHDF1/2/3, YTHDC1/2, IGF2BP1/2/3, HNRNPA2B1, HNRNPC, HNRNPG among others) [19, 20, 101, 110, 111]. In general, m6A RNA modification sites tend to be in the termination codons, the 5′ cap structure, and at the 3′- and 5′- untranslated region (3′-UTR and 5′-UTR) [93, 110–112].

In addition to gene expression regulation, m6A RNA methylation can influence cancer stem/initiating cell pluripotency, cancer cell differentiation and proliferation, cancer cell migration and metastasis, angiogenesis, tumor microenvironment, or immune regulation [101, 102, 113, 114]. In experimental and clinical studies, aberrant expression of m6A RNA regulators promotes tumorigenesis. However, some researchers also describe the tumor suppressive function of m6A regulators [99, 106, 107, 112, 115–120].

#### N1-methyladenosine

N1-Methyladenosine (m1A) was first identified in the total mixed RNA samples in 1961. As described in 1968, m1A can rearrange into m6A under alkaline conditions [121]. However, the functional research of m1A has become the scope of interest only in the last few years. Specifically, m1A modification involves adding an active methyl group from the donor to the nitrogen atom in 1st position of the adenosine in RNA [106]. Several m1A RNA writers (TRMT6, TRMT61A, TRMT10C, Trmt61B, RRP8), m1A erasers (ALKBH1, ALKBH3, FTO), and m1A readers (YTHDF1, YTHDF2, YTHDF3, YTHDC1) have been described [122–125]. Besides, m1A is highly enriched within the 5′-UTR or selectively at the start codon of transcripts [123, 126–130].

Regarding ncRNAs, m1A is a well-known modification in tRNA, rRNA, and lncRNA [128, 129, 131]. Current cancer research highlights the potential of m1A regulators to promote and sustain cancer cell proliferation, migration, and invasion to affect metabolic heterogeneity in cancer patients [124, 132–134]. Moreover, m1A modification patterns can predict cancer patient survival, stage, and grade. In the context of an immunotherapeutic strategy for cancer patients, m1A modification can have an essential role in shaping the immune microenvironment [135].

#### 5-methylcytosine

5-Methylcytosine (m5C) modification, firstly discovered in the 1970s in ribosomal (rRNA) and transfer (tRNA) RNA, occurs by methylation of RNA at the position of the 5th atom of cytidine residues [106, 136]. Notably, m5C modification can also fulfill different functions depending on the RNA subtype. For example, m5C is probably important for the nuclear export of mature mRNA [137]. Also, m5C can regulate tRNA structure and stability, or translation efficiency and accuracy, or can affect translational readthrough of termination codons in rRNA [138, 139]. The group of enzymes responsible for m5C modification of RNAs includes several types of m5C methyltransferases, namely members of the NOL1/NOP2/SUN domain (NSUN) family (NSUN1, NSUN2, NSUN3, NSUN4, NSUN5, NSUN6, NSUN7), further DNMT2, and TRDMT1. Besides, m5C erasers are represented by TET2, and m5C readers include YBX1 and ALYREF [125, 140]. m5C is preferentially accumulated around the translational start codons and 3′-UTRs of transcripts [141, 142].

Importantly, m5C modifications occur also in other types of ncRNAs, for instance, in lncRNAs, vault RNAs (vtRNAs), enhancer RNAs (eRNAs), or small Cajal body-specific RNAs (scaRNAs) [143]. Based on recent oncological studies, m5C RNA modifications possess diverse and extensive scopes of action. Specifically, m5C has oncogenic potential and can promote cancer progression, cancer cell migration, and metastasis and induce chemoresistance to anticancer therapy by methylation of various ncRNAs. Moreover, m5C significantly correlated with poor prognosis in cancer patients [112, 125, 140, 144–149].

#### 7-methylguanosine

7-Methylguanosine (m7G), characterized by methylation of guanosine on position N7, was first detected in 5′ caps of eukaryotic mRNA and subsequently internally in tRNA and rRNA [150–152]. More recently, m7G has been identified internally in miRNA precursors and mature miRNA, mRNA, and lncRNAs [153–156]. In mammals, several m7G regulators mediate m7G methylation of various RNAs—RNMT/RAM methyltransferase complex in mRNA, METTL1/WDR4 complex in tRNAs, and WBSCR22/TRMT112 complex in rRNA [157–159]. However, the research on identifying other specific m7G regulators is still limited. The primary role of m7G modification within mRNA is to sustain the translation process, in contrast with m7G modification within rRNA in which the effect on translation is weak. Moreover, m7G modification within mRNA is dynamically regulated by changes in stress conditions [159, 160]. In tRNA, m7G maintains the structural integrity of tRNA [161].

METTL1 (respectively the METTL1/WDR4 complex) has shown pro-oncogenic and tumor-suppressive activity within oncological research. The depletion or overexpression of METTL1 significantly affected the viability, proliferation, migration, and metastasis of various tumor cell types [155, 162–165]. Furthermore, METTL1 was crucial in regulating resistance to certain chemotherapeutic drugs such as 5-fluorouracil or cisplatin [166, 167]. In the clinical study of Tian QH et al. [168], METTL1 downregulated the tumor suppressor gene *PTEN*. Moreover, mainly within the integrated analysis and predictive models, METTL1, in combination with aberrant expression of other m7G regulators and various m7G-related RNAs, is correlated with poor prognosis of cancer patients [162, 168–172]. Significant tumor suppressive potential was also recorded in the case of other m7G regulators, i.e., WBSCR22 and TRMT112. Their overexpression significantly suppressed the proliferation, migration, and invasion of canscer cells in experimental models of pancreatic cancer [173].

#### 2′-O-methylation

2′-O-methylation (Nm or 2′O-Me in which N stands for any nucleotide) was first discovered in tRNA and rRNA in the 1960s [174]. Nm has also been found in mRNA, snRNA, and small non-coding RNAs such as siRNA, piRNA, and miRNA [175–183]. This type of methylation modification has not yet been found in lncRNAs. Recently, Wu H et al. [179] informed about the important regulatory role of lncRNA ZFAS1 in promoting 2′-O-Me modification in colorectal carcinogenesis. Notably, Nm is characterized by methylation of ribose at 2′-OH group and occurs in all four types of canonical nucleotides (i.e., Am, Gm, Um, and Cm) but also in other modified (non-canonical) nucleotides (i.e., Im and ψm) [175]. Two alternative enzymatic mechanisms can form nm modification. First, by stand-alone protein enzymes (stand-alone methyltransferases), or second, by a complex assembly of proteins (fibrillarin, or FBL) associated with snoRNA guides (sno(s)RNPs) (the box C/D (sno(s)RNPs)) [176]. Nm regulators include human methyltransferase CMTR1, capable of modifying the mRNA cap’s first transcribed nucleotide [184, 185]. FBL is the only known snoRNP 2ʹ-O-methyltransferase [186]. However, we know no erasers and readers have been discovered and studied for Nm yet. Nm was observed in the 5′ cap of mRNA, internally in the coding DNA sequence, further in the decoding or peptidyl-transferase centers within rRNA, or at the periphery of the ribosomal subunits [185–187]. Ribose methylation represents dynamic modification with a significant impact on RNA structure and stability regulation. Indeed, ribose methylation increases the hydrophobicity of RNA molecules, thereby protecting them from nuclease activity, alkaline hydrolysis, and oxidation [188]. Furthermore, ribose methylation affects mRNA splicing and translation, interactions of RNA with proteins or other RNAs, and the immune response of organisms [185].

Aberrant FBL expression can significantly affect rRNA methylation, thus ribosome biogenesis and function, protein synthesis, and cell proliferation that can subsequently result in cancer development [186]. Marcel V et al. [189, 190] have shown that FBL expression is under the direct control of p53, which acts as a repressor of FBL and therefore prevent the enhancement of the translation of various oncogenes. Moreover, alterations in box C/D snoRNA expression levels can also affect the process of carcinogenesis, for example, by promoting the stemness phenotype and proliferation of cancer cells or by their utilization for the prediction of cancer patient survival [191–205].

## The relationship between genome-wide methylation profiling and lncRNAs expression—the results from the most current BC studies

BC is a highly biologically and clinically heterogeneous disease characterized by histological and molecular diversity, distinct treatment responses, and prognostic patterns. Therefore, identifying reliable and highly informative diagnostic and prognostic BC biomarkers and therapeutic targets is highly required. DNA methylation biomarkers with diagnostic, prognostic, and predictive power significantly linked to BC or epigenetic therapies of BC focusing on the therapeutic effects of DNA methyltransferase (DNMT) inhibitors are in ongoing clinical trials [73, 83, 206–214]. Currently, as we mentioned above, several epigenetic studies are available regarding the relationship between the methylation status of DNA, RNA, or histones and the expression of various RNAs as biomarkers in the diagnosis and prognosis of several types of cancer, including BC as well as targets in personalized anticancer therapy [85, 104, 169, 215–222]. However, none of the studied RNA methylation biomarkers or epigenetic BC therapies that target DNA, RNA, or histone methylation in the context of various RNAs’ expression and function has not been approved for clinical use. Therefore, to deepen the current knowledge, we decided to summarize and discuss the results from the most recent BC studies (from 2017 to 2023) dealing with the relationship between genome-wide methylation profiling and lncRNAs. Several modes of interaction between lncRNAs and methylation modifications in BC have been described: (1) lncRNAs can be regulated by DNA methylation, DNA methylation negatively correlates with lncRNA expression; (2) DNA methylation can be regulated by lncRNAs that either recruit DNMTs or regulate the binding status of DNMTs; (3) lncRNAs can either regulate histone methylation or histone methylation can affect lncRNAs; and (4) lncRNAs can either regulate various type of RNA methylation or lncRNAs may themselves undergo specific methylation modifications [223]. Here, we focus on all the aspects mentioned above of the relationship between lncRNAs and methylation modifications in BC. Since many authors within more complex studies supplemented the analysis of BC tissue high‐throughput sequencing data with results from in vitro or in vivo experiments, we decided to divide corresponding BC studies into the following subsections based on the type of studied interaction.

### DNA methylation and lncRNAs

Several BC studies have shown that the decrease of DNA methylation levels upregulated the expression of oncogenic lncRNAs. On the contrary, increased DNA methylation levels downregulated the expression of antitumor lncRNAs. Wang Z et al. [224] observed upregulated lncRNA EPIC1, due to a promoter CpG island hypomethylation. At the same time, this overexpression was associated with BC cell cycle progression in in vitro and in vivo conditions. The authors further confirmed that EPIC1 overexpression was associated with significantly poor survival in luminal B BC patients. Another study showed that lncRNA HUMT could be upregulated by promoter hypomethylation that promotes lymphangiogenesis and metastasis by activating FOXK1 and increasing VEGF-C expression in TNBC.

Moreover, the higher level of HUMT was associated with poorer clinical prognosis in patients with TNBC [225]. Pangeni RP et al. [226] analyzed two (among others) epigenetically dysregulated genes coding two long intergenic non-coding RNAs (*RP11-713P17.4* and *CTD-2023M8.1*) in breast-to-brain metastases (BBM). Compared to normal breast tissues and primary breast tumors, *RP11-713P17.4* was hypermethylated, whereas CTD-2023M8.1 was hypomethylated in BBM. Moreover, some aberrant methylation patterns were found in tumor-free circulating DNA in the patient’s serum; however, a sample of serum should be taken during BBM biopsy. According to the authors’ conclusions, epigenetic dysregulation of *RP11-713P17.4* could be considered an early event in the process of BBM and could be used as BC prognostic marker. Another study showed that expression levels of GAS5 are commonly downregulated in cells and tissues of TNBC. However, targeted hypomethylation of GAS5 promoter increased the expression level of GAS5 in TNBC cells. Reducing TNBC cell proliferation and promoting TNBC cell apoptosis and chemosensitivity accompanied the increased expression of GAS5. These results indicate the role of GAS5 as a potential future candidate for TNBC treatment [227].

Similarly, the expression level of MEG3 in BC cells and tissues was poor, while the methylation rate of MEG3 was significantly increased. Targeted hypomethylation of MEG3 promoted the chemosensitivity of BC cells [228]. In addition, DNMT1 induced hyper-methylation of MEG3 promoter and facilitated the growth of BC via miR-494-3p/OTUD4 axis. On the contrary, the knockdown of DNMT1 enhanced MEG3 expression, upregulation of MEG3 downregulated miR-494-3p expression that also affected the expression of a miR-494-3p target OTUD4. Eventually, the authors concluded the inhibition of BC progression in vitro and in vivo [229]. Furthermore, tumor suppressor lncRNA HOTAIRM1 is downregulated and hypermethylated in BC tissues by DNMT1 and DNMT3A, promoting BC cell proliferation, migration, and metastasis.

Moreover, the authors confirmed that the downregulation of HOTAIRM1 could be a potential therapeutic target in BC due to its significant prognostic value [230]. LncRNA BLAT1 is significantly upregulated in basal-like breast cancer (BLBC). *BLAT1* promoter hypermethylation or hypomethylation may have an essential role in affecting the aggressive phenotype of BLBC cells. BLAT1 hypomethylation correlated with decreased overall survival in BLBC patients. Contrary, a depletion of BLAT1 significantly increased the apoptosis of BC cells [231]. Related findings from this subsection are summarized in Table 1.

**Table 1:** Methylation modification and its interaction with various type of lncRNAs in BC studies: DNA methylation → lncRNA

Long ncRNA	Expression in BC	Signaling pathway involved	Mechanism of action	References
EPIC1	↑	EPIC1/MYC	Promotion of BC cell cycle progression	[224]
HUMT	↑	HUMT/YBX1/FOXK1	Promotion of lymph-angiogenesis and metastasis in TNBC	[225]
RP11-713P17.4	not studied	Not studied	Hypermethylation of *RP11-713P17.4* gene in BBM	[226]
GAS5	↓	Not studied	Suppression of TNBC progression, reduction of TNBC cell proliferation, promotion of TNBC cell apoptosis and chemosensitivity	[227]
MEG3	↓	Not studied	Promotion of chemosensitivity of BC cells	[228]
MEG3	↓	DNMT1/MEG3/miR-494-3p/OTUD4	Supporting of the growth of BC cells	[229]
HOTAIRM1	↓	DNMT1 and DNMT3A/HOTAIRM1	Promotion of BC cells proliferation, clone formation, and invasion	[230]
BLAT1	↑	*BLAT1* promoter/BLAT1	Influencing the aggressive phenotype of BLBC cells	[231]

Abbreviations: ↑, increased, upregulated; ↓, reduced, downregulated; *BBM*, breast to brain metastases; *BCSCs*, breast cancer stem cells; *BLBC*, basal-like breast cancer

Research further confirms that methylation can affect lncRNAs, and various lncRNAs can regulate the process of DNA methylation during BC progression in several specific ways. Xu X et al. [201] recently analyzed the Cancer Genome Atlas BC high‐throughput sequencing data and BC study in vitro. The authors revealed that overexpression of lncRNA MAGI2-AS3, which acts as a cis-regulatory element to downregulate DNA methylation in the promoter region of MAGI2, inhibits proliferation and migration of BC cells and may be associated with a better prognosis of BC patient survival. Moreover, Wang HB et al. [232] described that LINC00518, which expression was significantly higher in BC tissues and cells, promotes the methylation of CDX2 by recruiting DNA methyltransferases and activating Wnt signaling pathway. This is ultimately promoting BC epithelial cell growth, proliferation, invasion, and epithelial-to-mesenchymal transition (EMT), and also the development of lymph node metastasis and suppression of apoptosis. In another study, authors showed that lncRNA H19 promotes tamoxifen resistance in estrogen receptor-positive (ER^+^) BC cells and autophagy in vitro and in vivo. The mechanism beyond H19 action affects the binding of DNMT3B and the Beclin1 promoter region by altering the SAH accumulation. The subsequent downregulation of the Beclin1 promoter methylation and promotion of tamoxifen resistance and autophagy of BC cells is modulated via the H19/SAHH/DNMT3B axis [233]. In addition, H19 regulated the expression of NAT1 in tamoxifen-resistant BC cells via the regulation of *NAT1* promoter methylation [234].

Furthermore, Li C et al. [235] uncovered that lncRNA MAYA and NSUN6 form an RNA–protein complex that methylates Hippo/MST1 resulting in MST1 kinase inactivation and YAP target gene activation, which consequently triggers BC osteoclast differentiation and bone metastasis development. Moreover, lncRNA 91H demonstrated oncogenic activity in vitro and in vivo; specifically, 91H promoted the aggressive phenotype of BC cells via regulating the expression of H19/IGF2 imprinting locus by masking the methylation site on the imprinting control center and the *H19* promoter [236]. In 2019, Miao H et al. [237] informed that lncRNA PYCARD-AS1 acts as a negative regulator of the pro-apoptotic gene *PYCARD* at both the epigenetic and translational levels in BC. PYCARD-AS1 facilitates DNA methylation of *PYCARD* promotor and H3K9me2 modification by recruiting DNMT1 and G9a, resulting in the silencing of *PYCARD* and disruption of the apoptotic process in BC cells in vitro. Moreover, the study showed that reactivation of *PYCARD* induced by the PYCARD-AS1-knockdown increased the susceptibility of BC cells to the cytotoxic agent paclitaxel. The analysis of BC samples, accompanied by experimental analysis in vitro and in vivo, described a novel HER2 subtype-specific lincRNA BCLIN25 that promotes mammary carcinogenesis by upregulation of ERBB2 expression via enhancing promoter CpG methylation of miR-125b. Downregulation of miR-125b led to the abrogation of ERBB2 mRNA degradation. The authors also provided a comprehensive landscape of molecular subtype-specific long intergenic noncoding RNAs, which could complement BC’s current molecular classification system [238].

Besides, lncRNA MIAT can bind to DNMT1, DNMT3A, and DNMT3B, promoting the methylation of CpG islands in DLG3 promoter and suppressing its expression. Moreover, DLG3 can bind to MST2, regulate LAST1, and prevent the nuclear translocation of YAP. Li D et al. [239] demonstrated that MIAT silencing inhibited the progression of BC by upregulation of *DLG3* and consequently led to the activation of mentioned Hippo signaling pathway. Moreover, the overexpression of lncRNA LINC00472 demonstrated the ability to suppress TNBC progression and inhibit proliferation, invasion, and migration of TNBC cells via regulation of DNA methylation. LINC00472 can significantly induce the methylation of *MCM6* promoter via recruiting DNMT1, DNMT3a, and DNMT3b, and thus reduce its expression. Subsequently, the inhibition *MCM6* expression led to the inactivation of the MEK/ERK signaling pathway and suppression of mammary cell cycle progression. Therefore, LINC00472-mediated epigenetic silencing of *MCM6* appears as a suitable therapeutic target for TNBC [240]. Another lncRNA TINCR, which was overexpressed in human BC and correlated with poor prognosis of BC patients, demonstrated in experimental conditions the ability to recruit DNMT1 to the miR-503-5p locus promoter, increasing methylation and suppressing the transcriptional expression of DNMT1.

Furthermore, TINCR acts as a ceRNA upregulated EGFR expression by sponging miR-503-5p. The study also revealed that TINCR could stimulate JAK2–STAT3 signaling downstream from EGFR and vice versa STAT3 enhances the transcriptional expression of TINCR [241]. Furthermore, TINCR reduced the effectiveness of immunotherapy against BC. Mechanistically, TINCR regulated the expression of USP20 and PD-L1 via ceRNA interaction and inhibition of miR-199a-5p transcription by promoting its methylation [242]. In the study of Wang Y et al. [243], lncRNA LINC00922 supported the progression of BC via *NKD2* silencing, activating Wnt signaling pathway, and promoting EMT, proliferative, invasive, and migratory capacities of BC cells. LINC00922 decreased the expression of *NKD2* by supporting the methylation of its promoter. Furthermore, Aini S et al. [244] showed that lncRNA SNHG10 could negatively regulate miR-302b methylation and that overexpression of lncRNA SNHG10 increased chemosensitivity of TNBC cells to doxorubicin via upregulation of miR-302b. In addition, the downregulation of lncRNA HOTAIR promoted the sensitivity of HER2^+^-resistant BC cells to trastuzumab when compared with sensitive cells, mechanistically via the upregulation of *PTEN* methylation levels, demethylation of TGF-β, and subsequent reduction of PI3K/AKT signaling pathway activity. Besides, increased PI3K/AKT activity is considered one of the leading factors responsible for the emergence of trastuzumab resistance in BC [245]. Moreover, Long Q et al. [246] showed that overexpression of lncRNA TATDN1 negatively regulated the expression of tumors suppressive miR-26b in TNBC cells, however positively affected methylation of miR-26b gene, thereby promoting the TNBC cell proliferation. Table 2 provides a detailed overview of the above-discussed findings.

**Table 2:** Methylation modification and its interaction with various type of lncRNAs in BC studies: LncRNA → DNA methylation

Long ncRNA	Expression in BC	Signaling pathway involved	Mechanism of action	References
MAGI2-AS3	↓	MAGI2-AS3/*MAGI2*/Wnt/beta-catenin	Inhibition of BC cell proliferation and migration	[201]
LINC00518	↑	LINC00518/*CDX2*/Wnt	Promotion of BC epithelial cell growth, proliferation, invasion, EMT, lymph node metastasis and suppression of apoptosis	[232]
H19	↑	H19/SAHH/DNMT3B	Promotion of tamoxifen resistance in ER^+^ BC cells trigger BC osteoclast differentiation and bone metastasis development	[233]
H19	↑	H19/*NAT1*	Promotion of tamoxifen resistance in BC cells	[234]
MAYA	↑	ROR1/HER3/MAYA	Stimulation of BC osteoclast differentiation and bone metastasis development	[235]
91H	↑	91H /*H19/IGF2*	Promotion of aggressive phenotype of BC cells	[236]
PYCARD-AS1	Not studied	PYCARD-AS1/*PYCARD*	Disruption of apoptosis of BC cells	[237]
BCLIN25	↑	BCLIN25/miR-125b/ERBB2	Promotion of HER2 BC	[238]
MIAT	↑	MIAT/*DLG3*/Hippo	Promotion of BC progression	[239]
LINC00472	↓	LINC00472/*MCM6*/MEK/ERK	Inhibition of progression and metastasis in TNBC	[240]
TINCR	↑	STAT3/TINCR/EGFR-feedback loop	Promotion of BC progression	[241]
TINCR	↑	STAT1/TINCR/miR-199a-5p/USP20/PD-L1	Reducing the effectiveness of immunotherapy against BC	[242]
LINC00922	↑	LINC00922/NKD2/Wnt	Progression of BC by promoting EMT, proliferative, invasive and migratory capacities of BC cells	[243]
SNHG10	↓	SNHG10/miR-302b	Suppression of chemoresistance of TNBC cells	[244]
HOTAIR	↑	HOTAIR/*PTEN*/TGF-β/PI3K/AKT	Promotion of trastuzumab resistance in HER2^+^ BC cells, reduction of their apoptosis, and promotion of their proliferative and invasion ability	[245]
TATDN1	↑	TATDN1/*miR-26b*	Promotion of TNBC cells proliferation	[246]

Abbreviations: ↑, increased, upregulated; ↓, reduced, downregulated; BBM, breast to brain metastases; BCSCs, breast cancer stem cells

### Histone methylation and lncRNAs

As mentioned above, lncRNAs can either regulate histone methylation or histone methylation can affect lncRNAs, which several BC studies have also confirmed. Firstly, lncRNAs can recruit polycomb proteins or methyltransferases associated with histone methylation of specific targets. For example, LINC00511 showed oncogenic function in ER-negative BC via interaction with EZH2 and recruiting PRC2 to mediate H3K27me3 modification in the promoter region of CDKN1B, which led to the suppression of CDKN1B expression. ER deficiency directly affected the expression of LINC00511, and high expression of LINC00511 indicated a markedly poorer prognosis in BC patients [247]. Another study demonstrated that lncRNA ROR supported BC progression by promoting H3K4 trimethylation of TIMP3 (via MLL1 recruitment) and enhanced its transcription levels. The expression levels of lncRNA ROR and TIMP3 were higher in BC tissues than in adjacent tissues. The results of this study provide evidence that lncRNA ROR can serve as a promising marker for BC prognosis and can be an important therapeutic target in BC therapy [248]. In addition, lncRNA UCA1 can support tamoxifen resistance of BC cells via regulation of the EZH2/p21 axis. UCA1 is associated with EZH2 suppressing the expression of *p21* through H3K27me3 on the *p21* promoter [249]. Also, the overexpression of lncRNA PHACTR2-AS1 promoted H3K9 methylation of rDNA by recruiting SUV39H1, thereby suppressing BC cell growth and metastasis. However, EZH2-induced silencing of PHACTR2-AS1 promoted ribosome synthesis and ribosomal DNA (rDNA) instability that, in turn, supported cancer cell proliferation and metastasis [250].

Furthermore, lncRNA HOTAIRM1 promoted the resistance of ER^+^ BC cells in vitro to tamoxifen via regulating HOXA1 expression through direct interaction with EZH2 and hindered deposition of H3K27me3 marks at *HOXA1* promoter [251]. LncRNA LINC02273 showed oncogenic potential and promoted BC invasion and metastasis in vitro and in vivo. Mechanistically, the hnRNPL-LINC02273 complex activated *AGR2* transcription and promoted BC metastasis by increasing H3K4me3 and H3K27ac levels around its promoter region [252]. Finally, lncRNA DANCR showed the ability to support EMT, cancer stemness, and inflammation in BC cells in vitro by promoting the binding of EZH2 to the *SOCS3* promoter, thereby inhibiting its expression [253]. Related findings from this subsection are summarized in Table 3.

**Table 3:** Methylation modification and its interaction with various type of lncRNAs in BC studies: LncRNA → histone methylation and Histone methylation → lncRNA

Long ncRNA	Expression in BC	Signaling pathway involved	Mechanism of action	References
LINC00511	↑	LINC00511/EZH2/PRC2/*CDKN1B*	Suppression of CDKN1B expression in ER^−^ BC	[247]
ROR	↑	ROR/MLL1/TIMP3	Support of BC progression	[248]
UCA1	↑	UCA1/EZH2/p21	Supporting of tamoxifen resistance of BC cells	[249]
PHACTR2-AS1	↓	EZH2/PHACTR2-AS1/Ribosome DNA	Suppression of the BC cell growth and metastasis	[250]
HOTAIRM1	↑	HOTAIRM1/EZH2/PRC2/HOXA1	Promotion of tamoxifen resistance in ER^+^ BC cells	[251]
LINC02273	↑	LINC02273/AGR2	Promotion of BC metastasis	[252]
DANCR	↑	DANCR/EZH2/*SOCS3*	Promotion of EMT, cancer stemness, and inflammation in BC	[253]
EPB41L4A-AS2	↓	ZNF217/EZH2/*EPB41L4A-AS2*	Promotion of BC progression	[254]
DLEU1	↑	DLEU1/*SRP4*	Promotion of BC progression	[255]

Abbreviations: ↑, increased, upregulated; ↓, reduced, downregulated; *BBM*, breast to brain metastases; *BCSCs*, breast cancer stem cells

Secondly, histone methylation marks on lncRNA can be associated with its activation or repression. Pang B et al. [254] first identified and validated the comprehensive landscape of tumor suppressor lncRNAs in BC tissues and subsequently selected lncRNA EPB41L4A-AS2 for further mechanistic investigation. Specifically, EPB41L4A-AS2 suppressed BC progression in vitro by upregulating the expression of RARRES1. Moreover, a high expression level of EPB41L4A-AS2 was associated with a favorable prognosis in BC patients. And finally, the authors showed that the progression of BC can be promoted by ZNF217 recruiting EZH2 to EPB41L4A-AS2 locus and suppressing EPB41L4A-AS2 expression by increasing H3K27me3 modification. Moreover, a decreased DNA methylation led to the upregulation of oncogenic lncRNA DLEU1 through increasing H3K4me3 and H3K27ac modifications in BC (in vitro and in vivo study supplemented by the TCGA and cohort data analysis). High DLEU1 expression correlates with a worse prognosis in BC patients. These findings indicate that epigenetic therapy targeting histone methylation modification in combination with DLEU1 target therapy may have the potential of an effective anti-BC strategy [255]. Related conclusions of this subsection are summarized in Table 3.

### m6A and lncRNAs

Although described as mRNA’s most frequent methylation modification, m6A is also commonly found in ncRNAs, including lncRNAs [256]. Several experimental studies demonstrated lncRNAs as less methylated than mRNAs, therefore assuming lncRNA methylation landscape differs from mRNA [62]. Indeed, lncRNAs can undergo m6A methylation modification, and some lncRNAs can participate in the modulation of m6A modification of the specific downstream target genes associated with BC. Specifically, METTL3 affected the LINC00675 sponge’s competitive endogenous RNA (ceRNA) network activity for miR-513b-5p through increasing m6A methylation modification. Besides, m6A methylation modification of LINC00675 did not affect lncRNA expression but enhanced the interaction between LINC00675 and miR-513b-5p and promoted BC repression [257]. The results of another study described eight m6A sites on HOTAIR. Among them, A783 was defined as consistently methylated. Besides, A783 interacts with m6A “reader” YTHDC1 enabling chromatin association and promoting high levels of HOTAIR expression and gene repression upstream of PRC2 complex, thereby promoting HOTAIR-mediated proliferation and invasion of TNBC cells [258]. Furthermore, Rong D et al. [259] showed that METTL3-induced LINC00958 upregulation promoted BC tumorigenesis via miR-378a-3p/YY1 axis necessary to regulate cell proliferation and apoptosis. In the study of Zhao C et al. [260], METTL3-induced upregulation of lncRNA MALAT1 regulated the progression of BC through the METTL3/MALAT1/miR-26b/HMGA2 pathway. Moreover, m6A modified lncRNA DLGAP1-AS1 and promoted adriamycin resistance in BC cells via WTAP/DLGAP1-AS1/miR-299-3p pathway [261]. Also, Sun T et al. [262] uncovered the oncogenic potential of LINC00942 (LNC942) and METTL14, which upregulated the expression and stability of two downstream target genes *CXCR4* and *CYP1B1* by promoting METTL14-mediated m6A methylation that subsequently led to accelerating BC cell proliferation, colony formation, and reduced BC cell apoptosis in vitro and in vivo. In addition, UCA1 regulated m6A modification of miR-375 by mediating METTL14 downregulation via DNA methylation. These results highlight miR-375 as poorly expressed in BC, and its expression positively correlated with METTL14 expression. Moreover, METTL14 mediated high SOX12 expression by m6A modification of miR-375 in BC in vitro and in vivo [263]. Furthermore, Zhu P et al. [264] demonstrated a significant role in the interaction between m6A and lncRNA in BC stem cells. Hypoxic lncRNA KB-1980E6.3 is upregulated in BC tissues and correlates with poor prognosis in BC patients. Specifically, KB-1980E6.3 recruited IGF2BP1 and maintained BC stemness and tumorigenesis by retaining c-Myc mRNA stability in vitro and in vivo. Table 4 summarizes related findings from this subsection.

**Table 4:** Methylation modification and its interaction with various type of lncRNAs in BC studies: m6A → lncRNA and lncRNA → m6A

Long ncRNA	Expression in BC	Signaling pathway involved	Mechanism of action	References
LINC00675	↓	METTL3/LINC00675/miR-513b-5p	Suppression of BC cell proliferation, migration, and invasion	[257]
HOTAIR	↑	YTHDC1/HOTAIR/PRC2	Promotion of proliferation and invasion of TNBC cells	[258]
LINC00958	↑	METTL3/LINC00958/miR-378a-3p/YY1	Promotion of BC tumorigenesis	[259]
MALAT1	↑	METTL3/MALAT1/miR-26b/HMGA2	Promotion of EMT, migration and invasion in BC	[260]
DLGAP1-AS1	↑	WTAP/DLGAP1-AS1/miR-299-3p	Promotion of adriamycin resistance in BC	[261]
LINC00942	↑	LINC00942/METTL14/CXCR4 and CYP1B1	Acceleration of BC cell proliferation, colony formation, and reduction of BC cell apoptosis	[262]
UCA1	↑	UCA1/METTL14/miR-375/*SOX12*	Progression of BC	[263]
KB-1980E6.3	↑	KB-1980E6.3/IGF2BP1/c-Myc	Maintenance of BCSC stemness under hypoxic conditions	[264]

Abbreviations**: **↑, increased, upregulated; ↓, reduced, downregulated; *BBM*, breast to brain metastases; *BCSCs*, breast cancer stem cells

### Other lncRNA methylation modifications in BC

Due to the lack of BC studies focusing on the interactions between lncRNAs and other types of methylation modifications such as m1A, m5C, m7G, or Nm, the association between these modifications and their effects on lncRNAs in human BC remains unclear and requires further research. Available research evidence provides no study on m1A- and/or Nm-related lncRNAs in BC. Some relevant studies deal with the relationship between m1A and various mRNAs in BC or m1A-related lncRNAs in other cancer diseases. Firstly, the earlier BC study by Singh B et al. [265] demonstrated the vital role of RNA demethylase FTO in the cell-based model of pan resistance in TNBC. The inhibition of FTO significantly suppressed the survival and/or colony formation of SUM149-MA TNBC cells compared to the control. At the same time, these effects were demonstrated via decreased demethylation of IRX3 mRNA and IRX3 protein synthesis. In another study, ALKBH3-induced m1A demethylation increased the CSF-1 mRNA stability in BT20 BC cells. The overexpression of ALKBH3 increased CSF-1 expression and invasiveness of BC cells without a significant effect on proliferation and migration [133].

On the other hand, Shi L et al. [131] realized m1A profiling of lncRNAs in human colorectal cancer (CRC). The authors revealed a significant difference in m1A distribution between CRC and adjacent non-tumorous tissues. They further determined downregulated lncRNAs along with m1A modification in CRC. And finally, they demonstrated the significant correlation between the unique distribution of m1A sites in lncRNAs with CRC signaling pathways. Similarly, in the case of m5C modification, the importance of the m5C-related lncRNAs was already studied in hepatocellular (HCC) and esophageal squamous cell carcinoma (ESCC). Still, we are not aware of a similar study in BC. In mentioned HCC study, m5C modification increased the stability of oncogenic lncRNA H19. Moreover, m5C-modified H19 demonstrated the ability to bond by G3BP1, which further led to MYC accumulation [266]. In the ESCC study, a novel NSUN2 methylated lncRNA NMR regulated tumor metastasis and drug resistance via NSUN2 and BPTF [144].

Within current BC research, scientists mainly focus on constructing BC prognostic signatures based on various lncRNAs associated with a particular type of methylation modification. Huang Z et al. [267] selected three BC-specific m5C-related lncRNAs (AP005131.2, AL121832.2, and LINC01152) that could have prognostic and predictive value in BC patients. Other authors recently analyzed the prognostic value of eleven m5C-related lncRNAs (AC002398.1, AL096701.3, AC073655.2, AL645608.7, AC244517.1, NDUFA6-DT, WEE2-AS1, AC090912.3, AL606834.2, AL136368.1, AC103858.2) and described an association between m5C-related lncRNAs and immune cell infiltration as well as chemotherapy drug sensitivity in BC patients [268]. Moreover, a comprehensive analysis provided potential m5C regulators in BC by a combination of expression, diagnosis, and survival analyses. Furthermore, the authors established the ncRNA–mRNA network accounting for the role of m5C regulators in BC in which several upstream potential lncRNAs of the five upstream potential binding miRNAs of m5C regulators (let-7b-5p, miR-195-5p, miR-29a-3p, miR-26a-5p, and miR-26b-5p) were predicted and analyzed. Among the examined m5C regulators, DNMT3B and ALYREF were significantly upregulated in BC samples. At the same time, their high expression indicated an unfavorable prognosis in BC patients and possessed the statistical abilities to distinguish BC tissues from normal breast tissues. Pathway analysis revealed that *VEGFA* and *EZH2* represent the most potential target genes in BC’s m5C regulators-related ncRNA–mRNA network. The upstream potential lncRNAs of studied miRNAs are listed in the supplementary material of the study [269]. Moreover, another BC prognostic signature was based on m7G-related lncRNAs. Huang Z et al. [270] identified eight m7G-related lncRNAs (BAIAP2-DT, COL4A2-AS1, FARP1-AS1, RERE-AS1, NDUFA6-DT, TFAP2A-AS1, LINC00115, and MIR302CHG) and Cao J et al. [271] nine m7G-related lncRNAs (LINC01871, AP003469.4, Z68871.1, AC245297.3, EGOT, TFAP2A-AS1, AL136531.1, SEMA3B-AS1, AL606834.2), which could serve as potential biomarkers and therapeutic targets of BC.

## Reciprocity of genome-wide methylation status and lncRNA patterns in BC: concluding remarks on potential benefits to the 3 PM approach

The application of genome-wide methylation analyses strongly contributes to understanding lncRNAs-associated pathomechanisms in BC development and progression. It presents a powerful diagnostic, prognostic, and therapeutic tool in the context of 3P medicine [272]. Stage-specific lncRNA expression patterns are instrumental for the differential diagnostics, targeted prevention, and treatment tailored to the individualized patient profiles. On the other hand, genome-wide epigenetic shifts individually analyzed for BC patients are an essential indicator for an accurate prognosis and targeted preventive strategies [273]. Genome-wide methylation analyses and stage-specific lncRNA patterns synergistically increase the predictive power of BC diagnostics and the efficacy of the targeted anti-cancer therapy [274]. To this end, the role of the advanced 3 PM approach is to distinguish the “driver” genomic methylation events from their “passenger” functions, which is considered crucial for personalized treatment algorithms in BC management [275–277].

## Mitochondrial health as the prominent example of comprehensive epigenetic regulations involving methylation and lncRNA-specific patterns highly relevant for primary and secondary BC care: a proposal for future PPPM approach

### Predictive diagnostics

Mitochondrial health quality controls and regulates cellular, organ, and organismal metabolism [278]. Mitochondrial plasticity (fission, fusion, mitophagy) is crucial to integrate environmental and internal signals and govern an adequate reaction in physiological bioenergetics and multi-functional response to diverse stress stimuli. In contrast, mitochondrial dysfunction and burnout under severe medical conditions create extensive oxidative stress causing epigenetic dysregulation reflected in shifted DNA methylation and histone modification. To this end, oxidative and nitrosative stress provoked by injured mitochondria is a powerful systemic predictor of the health-to-disease transition reflected in corresponding health condition-specific multi-omic patterns well detectable in body fluids such as blood and tears [279, 280]. Corresponding systemic molecular signature is associated with an impaired immune function and cross-talking miRNAs and lncRNAs, exosomal ncRNA communication to cells and tissues [281], and increased extracellular presents of mtDNA fragments that are instrumental for predictive diagnostics with a great potential to reverse a disease development at the stage of health-to-disease transition [280].

### Mitochondria health control is pivotal for the targeted primary and secondary BC care

Under the influence of endogenous and environmental agents such as xenobiotics (environmental pollutants and heavy metals) and therapeutic drugs, the methylation status of mtDNA is changing, which may result in altered bioenergetics and decreased ATP production, metabolic disorders, accelerated aging, and related pathologies such as chronic degenerative processes and cancers including metastatic breast cancer malignancies. It is abundantly described that epigenetic regulation of mtDNA and mitochondrial proteins allows for cross-talking between the nucleus and mitochondria, orchestrating and maintaining cellular health and physiologic mitochondrial homeostasis [282]. To this end, methylation occurs in mitochondria via DNA methyl-transferases identified in the organelle and regulated via long- and short-noncoding RNAs [282].

Extensive evidence is provided for regular body exercise as an effective risk mitigation measure applied to primary (disease predisposition and development) and secondary (improved individual outcomes in treated breast malignancies) BC care [7]. Accumulated knowledge demonstrates that all three components are involved in protective mechanisms: increasing the population of healthy mitochondria and epigenetics and lncRNA regulation that functions reciprocally. To this end, the role of long non-coding RNA taurine-upregulated gene 1 (TUG1) was recently investigated, which interacts with PGC-1alpha in regulating a transcriptional response to exercise in skeletal muscle. TUG1 expression was upregulated and positively correlated with an increased PGC-1alpha expression in human skeletal muscles associated with mitochondrial calcium handling and improved myogenesis after a single exercise session. In contrast, Tug1 knockdown in mouse myotubes led to impaired mitochondrial respiration and morphology [283].

Furthermore, mitochondrial oxidative phosphorylation (OXPHOS) regulates metastatic disease. Arginine and lysin methylation of MRPS23 promote breast cancer metastasis by regulating OXPHOS, which opens the door for new therapeutic options based on mitochondrial epigenetic regulation [284].

Finally, metformin treatment, considered anti-diabetic and anti-cancer protection, employs epigenetic regulation. Metformin and a mitochondria/complex I (mCI)-targeted analog of metformin promote DNA methylation in non-cancerous, cancer-prone, and metastatic cancer cells by decreasing S-adenosylhomocysteine (SAH) being capable of reprogramming the DNA methylation machinery [285].

## Phenotyping as the screening tool for a cost-effective 3 PM approach

To increase the overall efficacy of applying PPPM to BC management, phenotyping has been proposed as a screening tool of great clinical utility [286, 287]. To this end, the below listed phenotypes are recommended candidates for innovative screening programs and predictive approaches in the population. Their relevance for epigenetic dysregulation and BC predisposition is justified in recent publications.

**Compromised mitochondrial health** is strongly associated with BC development and progression into metastatic disease. As stated above, oxidative and nitrosative stress provoked by injured mitochondria is a powerful systemic predictor of the health-to-disease transition reflected in corresponding health condition-specific multi-omic patterns well detectable in body fluids such as blood and tears [279, 280]. Corresponding systemic molecular signature is associated with an impaired immune function and cross-talking miRNAs and lncRNAs, exosomal ncRNA communication to cells and tissues [281], and increased extracellular presents of mtDNA fragments that are instrumental for predictive diagnostics with a great potential to reverse a disease development at the stage of health-to-disease transition [280]. Population screening for compromised mitochondrial health is recommended for primary care.

**Increased blood plasma homocysteine** (Hcy) is associated with metabolic and DNA methylation shifts. Subtile changes in Hcy concentration by 13–14 µmol/L (against 11 µmol/L breast cancer-free controls) are associated with an increased risk of BC, T stage of the disease, and lymph node metastasis in BC patients [288]. H19 lncRNA inhibits S-adenosyl homocysteine (SAH) hydrolase, the only mammalian enzyme capable to hydrolise SAH. In turn, SAH inhibits S-adenosyl methionine (SAM)-dependent methyltransferases methylating key biomolecules such as DNA, RNA, proteins, lipids, and neurotransmitters. Consequently, genome-wide methylation status is shifted in individuals with altered Hcy patterns—a critical sub-population recommended for the screening. Primary BC prevention by dietary folate supplement effectively normalizes a slight increase in Hcy concentration [289, 290].

**Endothelial dysfunction** is linked to the diabetes type 2 phenotype (DMT2) and Flammer syndrome (FS)—both predisposed to increased risk of breast cancer with poor outcomes [286, 291–293]. In both phenotypes, endothelin-1 (ET-1) is upregulated, leading to imbalanced vasoconstriction, ischemic-reperfusion events, metabolic impairments with cascading complications, aging and related pathologies, cardiovascular diseases, neurodegenerative pathologies, and aggressive malignancies [294]. Specifically in DMT2, lncRNAs are involved in the glucose-induced transcriptional upregulation of ET-1 via hypomethylation in the proximal promoter and 5′ UTR/first exon regions of the EDN1, while knocking down specific lncRNA panels suppresses epigenetic upregulation of ET-1 [295]. Furthermore, depending on the individual phenotype and environmental conditions and consequently being driven by epigenetic regulation mechanisms, the ET-1 axis affects the invasiveness of metastatic BC [296–299]. Both DMT2 and FS phenotypes are contextually recommended for population screening related to ET-1-associated BC risks.

An advanced health policy should essentially consider the above-provided recommendations in order to improve the overall BC management.

## Abbreviations

BC: Breast cancer
IARC: The International Agency for Research on Cancer
lncRNAs: Long non-coding RNAs
ncRNAs: Non-coding RNAs
3P medicine: Predictive, preventive, and personalized medicine
